# Development and validation of traditional & complementary medicine (TCM) scales for nurses: Using structural equation modelling (SEM)

**DOI:** 10.1186/s12906-019-2733-z

**Published:** 2019-11-21

**Authors:** Hsiao-Yun Chang, Chia-Lun Lo, Yun-Ying Hung

**Affiliations:** 1 School of Nursing, Fooyin University, 151 Jinxue Road, Daliao District, Kaohsiung City, 83102 Taiwan; 2 Department of Health-Business Administration, Fooyin University, Kaohsiung, Taiwan; 3 Department of Nursing, Chung Hwa University of Medical Technology, Tainan, Taiwan

## Abstract

**Objectives:**

This study aimed to develop and validate scales to assess attitudes towards patient’ s use of TCM (APUTCM) and to measure a communicative competence in TCM (CCTCM) among nurses.

**Methods:**

The instrument development process was conducted from Sep 2013 to Jul 2014, using the following steps: 1) item development; 2) internal review and refinement; 3) face and content validation; 4) instrument administration to a development sample; and 5) evaluation of validity and reliability. A convenience sample was used to recruit registered and advanced practice nurses who worked in different regions throughout Taiwan. A total of 755 respondents completed the online questionnaire. Statistical analyses were performed using the software of SPSS Version 21.0 and Analysis of Moment Structures (AMOS) version 24.0.

**Results:**

The scale-level indexes (S-CVI) of content validity for both scales were over 80%. The reliabilities for the 13-item APUTCM scale and for the five-item CCTCM scale were 0.88 and 0.84, respectively. The model suitability for both scales was assessed, and the findings revealed suitable parameters for all indicators: GFI = 0.954, AGFI = 0.932, CFI = 0.959, RMSEA = 0.62, and chi-square/df = 3.891 for APUTCM; and GFI = 0.992, AGFI = 0.969, CFI = 0.992, RMSEA = 0.63, and chi-square/df = 4.04 for CCTCM. The convergent and divergent validity of scores on both scales provided evidence in the expected direction.

**Conclusion:**

This scale development study provides preliminary evidence that suggests that the 13-item APUTCM and the five-item CCTCM are reliable and valid scales for assessing attitudes toward patient’s TCM use and communicative competence in TCM.

## Background

The definition of Traditional & complementary medicine (TCM) by the World Health Organization (WHO) refers to a broad set of health care are knowledge, skill, and practices approaches not fully integrated into the dominant health-care system [1]. TCM is an umbrella term that encompasses various health services that do not belong to conventional medicine, such as traditional medicine, complementary medicine, alternative medicine, herbal medicine, integrative medicine, and so on. The prevalence of TCM providers use among adults was 26.4% from 32 Countries and over 50% usage rate in Asian countries was found, such as Taiwan, Philippines and Korea [2]. The popularity of TCM use among patient population is also respectively high. With 94% of patients presenting at the emergency room were TCM users and 80.9% of them do not inform their healthcare professionals about their TCM use which may lead to an increase in the adverse effects of TCM-drug interaction [3]. The common reasons for the non-disclosure of CAM use among patients with arthritis are because they are experienced by unsupportive or negative attitudes from healthcare professionals and lack of knowledge about TCM [4]. In fact, most nurses (73%) do not ask patients about TCM use [5]. As noted in the literature review, we have found globally that many nurses have a lack of TCM knowledge, education, training, institutional support, and resources that are necessary for TCM discussions with patients [6].

The WHO Traditional Medicine Strategy: 2014–2023 has aimed to promote the safe and effective use of TCM by regulating, researching, and integrating these practices into healthcare systems [1]. Given the popularity of TCM use among patients, the less discussion on TCM use between patients and healthcare professionals and the potential adverse TCM-drug interaction, it has gradually resulted in the communication of TCM becoming an important issue among health care professionals. In order to achieve competence in TCM communication, it is essential to identify healthcare professionals’ core values and belief systems that underpin attitudes towards patient’s TCM use. As one’s belief (knowledge) is an internal perception that leads to the development of attitudes that result in the expression of words and direct behaviours, for example, motivation, engagement and intention to complete the task [7].

An inconsistency of scales regarding beliefs, attitudes, and behaviours towards TCM has been observed between studies. Most researchers used a checklist of TCM modalities to assesses nurses’ perceptions, belief, and attitudes, knowledge, clinical usefulness, and willingness to recommend for patients; or using yes/no responses to several statements regarding their behaviours and communicative behaviours [6]. These questionnaires seldom investigate reliability and validity, which cannot be classified using a psychometric scale. The earliest scale related to attitudes towards TCM was developed by Schneider, Meek, & Bell [8] that included a 29-item “integrative medicine questionnaire (IMAQ)” with two subscales, of “openness to new ideas and paradigms” and “value of both introspection and relationship to patient.” The purpose of this study was to understand healthcare professionals’ willingness to use TCM in their practice. In 2005, Schmidt et al. [9] design a scale that measured attitudes toward TCM and included three subscales: 1) “attitudes toward holism”; 2) “attitudes toward the effectiveness of TCM”; and 3) “attitudes toward introspection and doctor-patient relationship.” Unfortunately, the internal consistency reliability remained low (0.41–0.71), and the comparative fit index (CFI) was less than 0.9. In 2011, Abbott et al. [10] re-developed this scale, which was named the “Complementary, Alternative, and Integrative Medicine Attitude Questionnaire (CAIMAQ).” The items on this scale were based on the IMAQ; however, the core concepts of IMAQ are more focused on patient care, rather than a holistic TCM approach. These measurements include the ability of the body to heal, healthcare professional-patient relationships, shared medical decisions, health and well-being for patients, and preventive medicine.

As noted in the above studies, inconsistencies exist in the operational definitions of each variable, in the answer patterns, in the true value of healthcare professional attitudes, and in the communication of TCM. In addition, these earlier studies were generally performed at single sites, used self-developed instruments, and did not report reliability and validity information. To the best of our knowledge, there is no scale to assess healthcare professionals’ competence in TCM communication; the available literature regarding TCM communication is limited to the communication rate of TCM and the reasons for the barriers to TCM communication. Nurses have highest employment in healthcare occupations [11] and usually, nurses as a front-line role of healthcare providers whose behaviour is a major factor influencing patients’ healthcare experience and their perception in the quality of healthcare. Given the scarcity of studies in this area, both at the national and international levels, the purpose of this study is to build scales related to TCM, including a scale that measures nurses’ attitudes towards patients’ TCM use and their communicative competence in TCM use with patients.

## Materials and methods

### Study design

This study used an instrument development methodology to construct a psychometric scale. The process of instrument development was conducted following a set of guidelines outlined by DeVellis [12] and Henderson et al. [13], which included: 1) item development; 2) internal review and refinement; 3) face and content validation; 4) instrument administration to a development sample; and 5) evaluation of validity and reliability. This study used this process to construct a scale that measures nurses’ attitudes towards patients’ use of TCM (APUTCM), which were based on the theoretical underpinning of the tri-component attitude model. This model captures the underlying attitude dimensions to better explain or predict behaviour, including cognition, affect, and conation [14]. We also developed a valid communicative competence scale that assessed nurses’ ability in communication of TCM with patients. This scale was termed the “Communicative Competence in TCM (CCTCM)” scale.

### Scale development: item development

The generation of a large pool of items that were candidates for scale inclusion involved two parts: 1) the use of a previous focus group data set that was related to bridging of the communication gap between patients and nurses towards discussing TCM use; and 2) a thorough literature review on nurses’ knowledge, attitudes, and ability to communicate the risks and benefits of TCM [6]. The first step was to map and interpret the coded data of focus groups that was recruited from nursing students who were enrolled in a post-registration Bachelor of Nursing program (Post-RN) or a master’s degree nursing program. For concept mapping, data were generated from the participant’s own words, and the items included beliefs, values, attitudes, behaviour, communication skill, assessment, implementation, accountability, patient right, usage, production, and therapeutic effects.

Secondly, extensive and comprehensive searches were conducted using the following databases: MEDLINE, CINAHL, PsycINFO, PubMed, Cochrane library, Evidence-based Practice Institute and Airiti library (Chinese). The thematic analysis of articles was independently performed by two researchers. To compare and contrast between studies, critical appraisal and data extraction of the characteristics of the included studies were presented in tabular form. Following the analysis of focus group data and the literature review, the congruent core concepts that were essential to the APUTCM and CCTCM scales were identified with two pools of items, which helped researchers develop an instrument that relates to healthcare professionals’ cognition, affect and conation, and a competence scale for communicating about TCM with patients.

### Scale development: internal review and refinement

Once the conceptual issues were determined, a panellist of experienced researchers from clinical and academic backgrounds had a brainstorming session to design components from the item pool in response to the purpose of this study and to review items to verify that the wording that reflected the concept about which information should be sought. Four measures (readability, length of items, reversed items, and the format of measurements) were considered in the refinement process. The APUTCM scale contained 41 items and the CCTCM scale had 17 items.

### Scale development: face and content validation

After generating a pool of suitable items and selecting a response format for the items, the specific meaning of each item was examined to establish face validity. An open discussion was performed for each item separately, with 10 registered nurses from different units and hospitals. Participants examined whether the words in the item reflected the intended meaning. Following face validation, the instruments were evaluated for content, to ensure that the instruments had the appropriate items for the measured construct. The content validity indices (CVIs), which included the item-level CVIs (I-CVIs) and the scale-level index (S-CVI), were computed using item relevance ratings, which were performed by content experts. Eleven item-level CVIs (33–66%) were lower than 75% and were discussed by the research team. Of these 11 items, we revised two and deleted nine due to familiarities with other items. The S-CVIs were 88% for the 36 items of the APUTCM scale and 84% for the 14 items of the CCTCM scale.

### Scale development: instrument administration to a development sample

A convenience sample was used to recruit registered nurses and advanced practice nurses who worked in different regions in Taiwan. To be eligible for this study, participants had to meet the following criteria: 1) over the age of 20; 2) more than a year of work experience as a nurse; and 3) employment in participating hospitals at time of data collection. Conventional recommendations for sample size in psychometric studies range from five to 10 respondents per scale item for exploration factor analyses [15]; and finally, 755 respondents participated in our study. This study was approved by the ethical committees (Fooyin University Hospital with the number of FYH-IRB-101-12-01-A) and informed consent to participate was obtained from all participants.

### Scale development: data analysis for reliability and validation

The statistical analyses were performed using the SPSS Version 21.0 software package for Windows (SPSS, Inc., Chicago, IL, USA), and Analysis of Moment Structures (AMOS) version 24.0 was used for confirmatory analysis through structural equation modelling (SEM). The demographic characteristics of the participants were described by descriptive statistics. The proportion who were in agreement about the appropriateness and accuracy of reflection to TCM communication concepts were calculated using the CVI. After content validation, the item analysis was used to select items for the psychometric scale. The internal consistency reliability was assessed using Cronbach’s alpha coefficient (α > 0.7) for each dimension of the scale, to ensure that the various items that measured the different constructs delivered consistent scores [16]. An exploratory factor analysis, which used principal axis factoring and varimax rotation to enhance the interpretability of the extracted factorial model, was used first to explore a relatively large set of items. The number of factors was determined by the Kaiser method of eigenvalues greater than one and the communalities of the Varimax orthogonal rotation. Small coefficients (values less than 0.4) were suppressed [15].

A confirmatory factor analysis and SEM were conducted using AMOS 24.0, to determine the constructing validity of the APUTCM and CCTCM scales. Structural equation models are widely used in empirical research to investigate relationships among variables. We used the maximum likelihood estimation (MLE) robust extraction method to estimate parameter estimation. To evaluate the global fitting quality, five indices were used to assess model fit: 1) ratio of chi square and degrees of freedom: if the value of the equation was equal or smaller than two, the fit was perfect, the fit was good for values lower than five, and the fit was unacceptable for values greater than five; 2) goodness of fit index (GFI), comparative fit index (CFI), and the adjusted goodness of fit index (AGFI) recommended values greater than 0.9, and values greater than 0.90 were considered to represent good fits; 3) root mean square error of approximation (RMSEA) recommended values under 0.08 to indicate perfect fits [17].

The association between nurses’ attitudes towards TCM and their communicative competences in TCM was tested for the convergent validity. The discrimination validity of TCM communicative competence was used to examine the differences between nurses with and without TCM education.

## Results

### Demographic characteristics

A total of 755 respondents who were recruited from January to April 2014 participated in this study. Table 1 shows the demographic characteristics of the participants. There were 12 men and 743 (98.4%) women, ranging in age from 21 to 60, with an average age of 33 years. Approximately, 78.6% of the respondents had graduated with a Bachelor of Nursing degree. The majority of participants were single (53.7%), practicing Daoism (60.1%), had not received TCM education, had not used TCM in the past year (65.6%), and had not communicated TCM with patients (81.3%). Most of them practiced as registered nurses (*n* = 678, 89.8%) or clinical nurse specialists (*n* = 77, 10.2%) and had been working in clinical practice for a median of 10 years (ranging from 1 to 33 years).

**Table 1:** Participants’ demographic characteristics (*n* = 755)

Demographic characteristics	Mean	SD
Age	34	±7.19
Years of experiences	10.17	±7.07
	n	%
Gender		
Male	12	1.6%
Female	743	98.4%
Education		
Associate bachelor	162	21.4%
4-year bachelor	254	33.6%
2-year bachelor	298	39.5%
>Master degree	41	5.4%
Marriage		
Single	406	53.8%
Married	332	44.0%
Divorced	14	1.9%
Widow	3	0.4%
Religion		
Daoism	454	60.1%
Buddhism	103	13.6
Christian	66	8.8%
Others	132	17.5%
Employed position		
Nurse	678	89.8%
Clinical nurse specialist	77	10.2%
Frequency of Journal readings		
No	266	35.2%
1–2	388	51.4%
> 3	101	13.4%
Received TCM education		
No	445	58.9%
Yes	310	41.1%
Self-use of TCM		
No	495	65.6%
Yes	260	34.4%
Practice of TCM		
No	615	81.4%
little	140	18.6%
Perceived knowledge of TCM		
No	120	15.9%
little	384	50.9%
Some	184	24.4%
Medium	59	7.8%
High	7	1.0%
Frequency of TCM communication		
No	206	27.3%
little	373	49.4%
Sometime	121	16.0%
Often	50	6.6%
Always	5	0.7%

### Item analysis

The items with item-total correlation below 0.4 and above 0.7 were deleted due to either insufficient contribution to concept measurements or redundancies. After APUTCM scale and CCTCM scale item analysis, the item-total correlation values above 0.70 and below 0.40 were eliminated, which resulted in the 22-item APUTCM scale and the nine-item CCTCM scale.

### Reliability: internal consistency reliability

The scale was then tested for internal consistency reliability. Cronbach’s α was calculated for each scale to assess internal consistency. The α coefficient for the 22-item APUTCM scale was 0.93, and that for the nine-item CCTCM scale was 0.94. After the exploratory factor analysis, the internal consistency reliability was re-calculated using the α coefficient for the whole scale and its subscales. The 13-item APUTCM scale had a value of 0.88, with 0.84 for the cognitive component, 0.82 for the affective component 0.82, and 0.77 for the behavioural component subscales. The α coefficient of the five-item CCTCM scale was 0.84, with 0.81 for the sustainability and 0.67 for the subscales.

### Factor analysis: construct validity

The items that remained after the item analysis were further used to estimate construct validity by exploratory factor analysis (EFA) and confirmatory factor analysis (CFA). The EFA has a limitation at its subjectivity stemming level, which involves the many methodological decisions a researcher must make to complete a single analysis, and the accuracy of the results largely depend upon the quality of these decisions [15]. In this study, a principal component method (SPSS PAF Factor analysis) with iteration was performed. The number of factors chosen in ease case was determined by several conditions: (1) disregard of the rotated factors that had two or fewer variables; (2) disregard of the factors with loading scores below a certain threshold (γ < 0.4) for each factor.

After applying the EFA criteria described above, a factor analysis was performed for the 22 items of the APUTCM scale. The total number of factors was eliminated to enhance the simple structure criterion and increase the interpretability of the scale for complementary and alternative medicine selections. Three factors with eigenvalues exceeding one were retained. Seven items were excluded, and three factors that were labelled “cognitive component, affective component, and behavioural component”, with eigenvalues exceeding one, were retained. A total of nine items were excluded from the scale construction because of insufficient or dual factor loadings, and 13 items were included for the confirmatory factor analysis. For the nine-item CCTCM scale, two factors that were labelled “sustainability and performance”, with eigenvalues exceeding one, were retained, and four items were eliminated.

In the CFA session of this study, data were analysed using the SEM approach and AMOS tools. The SEM analysis includes the testing of theoretical hypotheses and relationships. The path diagrams that were used to represent the model were composed of circles or ellipses that represent unobserved variables. The single headed arrows represent the impact of one variable to another, and the double headed arrows represent the correlations between one variable and another. In this study, the APUTCM and CCTCM models that resulted from the exploratory factor analysis were tested, and we observed that all items had acceptable absolute skewness values. The dual model was further adjusted, considering the covariance of modification indices by AMOS. Table 2 list the critical ratios of the trajectories between the items of the two scales.

**Table 2:** Trajectories and critical ratios of the APUTCM and CCTCM scales

Trajectories	Estimate	Stand error	Critical ratio
APUTCM Scale			
Cognitive Component			
Perceived needs for increasing patient awareness of TCM	1.000		
Belief in TCM as a patient self-management strategy	0.925	0.064	14.498^***^
Perceived insufficient knowledge about TCM.	1.094	0.073	14.927^***^
Integration of TCM into conventional care to address patients’ needs	1.116	0.068	16.435^***^
Affective Component			
Understanding of TCM assistance in the care of patients with the needs	1.000		
Acceptance and respect of patient’s TCM use	1.161	0.063	18.329^***^
The right to use both CM and TCM for patients	1.243	0.064	19.277^***^
Respect of patients’ TCM choices in disease self-management	1.175	0.061	19.386^***^
Behavioural Component			
Familiar with their own specialist-related TCM	1.000		
Ability to evaluate the efficacy and safety of TCM	1.757	0.176	9.982^***^
Ability to search for trusted TCM information	1.934	0.188	10.271^***^
Ability to face TCM challenges with confidence	2.010	0.194	10.375^***^
Ability to assess and evaluate patient’s TCM use.	1.858	0.180	10.301^***^
CCTCM Scale			
Sustainability			
Receiving training to improve TCM communication skills	1.000		
Learning integrative medicine to facilitate communication with patients regarding TCM use	1.381	0.093	14.895^***^
Performance			
Answering TCM questions from evidence-based literature	1.000		
Feeling confidence in discussing TCM-related questions	0.902	0.046	19.627^***^
Listening patients’ TCM use in a respectful and open-minded manner	0.928	0.044	21.252^***^

***=*p*< .001

**Fig. 1:**
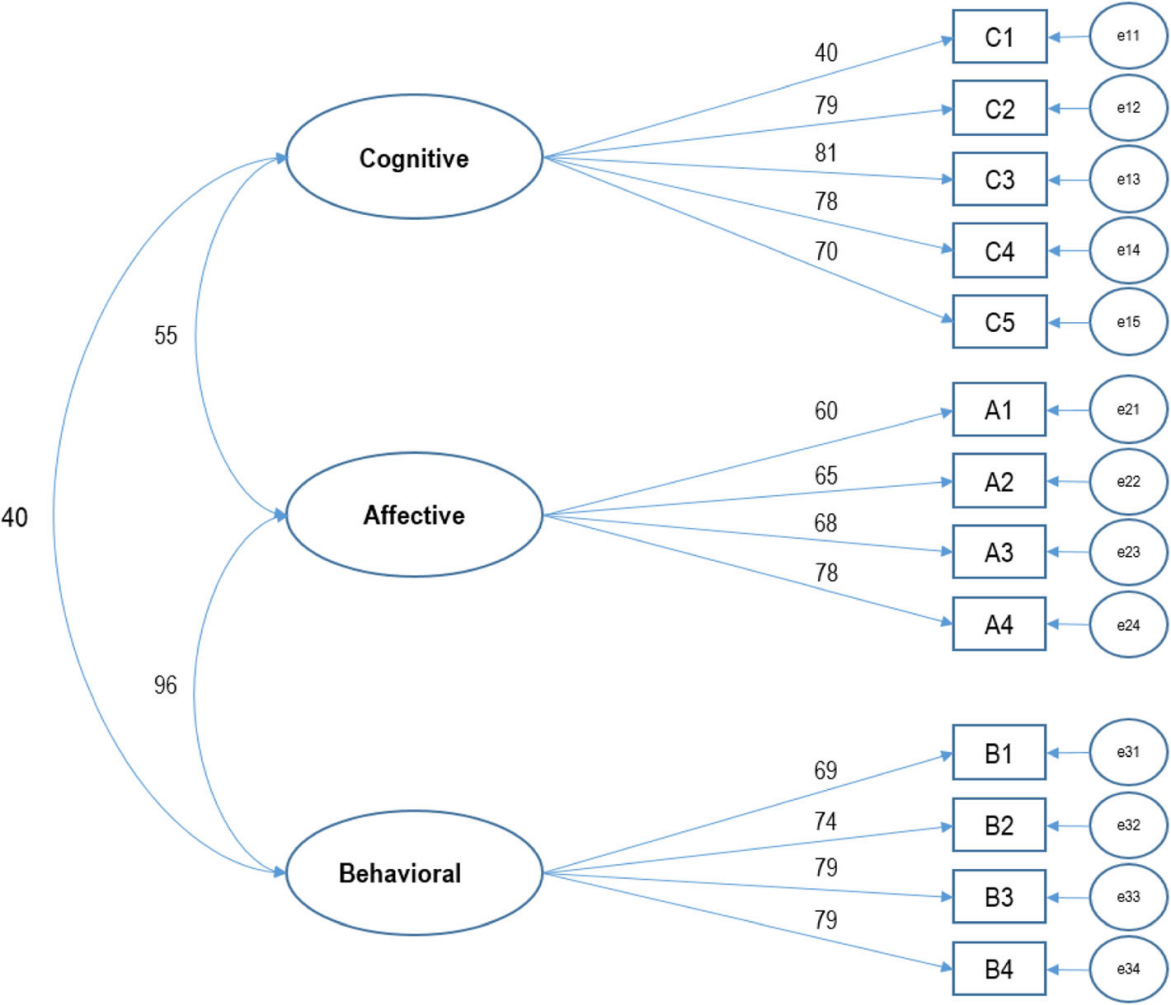
Adjusted model for APUTCM

After the measurement model (a confirmatory factor analysis model) was assessed to examine reliability and validity, the model suitability was also assessed. First, the final APUTCM model is shown in Fig. 1, which reveals that most items have factor weights greater than 0.60, with corresponding latent variables. The model revealed suitability for all indicators (GFI = 0.954, AGFI = 0.932, CFI = 0.959, RMSEA = 0.62, and chi-square/df = 3.891).

**Fig. 2:**
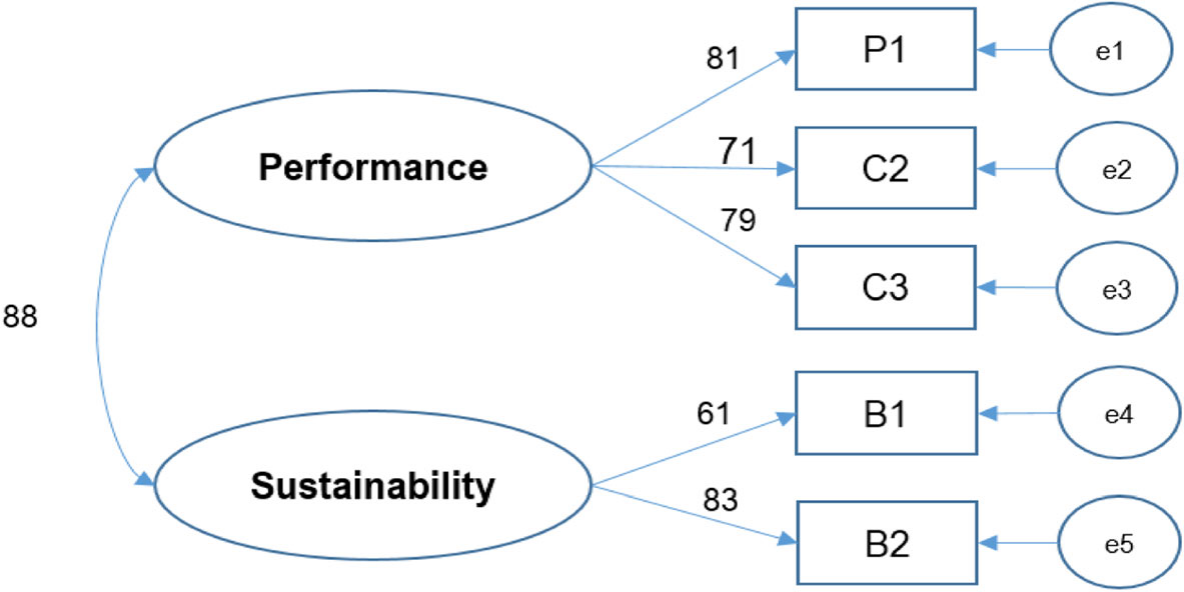
Adjusted model for CCTCM

The CCTCM model that was tested is shown in Fig. 2, and the results good values for all of the indices that were considered (GFI = 0.992, AGFI = 0.969, CFI = 0.992, RMSEA = 0.63, and chi-square/df = 4.04). There are some drawbacks associated with the ability of the chi-square test to show statistical significance, particularly for larger sample sizes. In this case, the indicator for the goodness of fit should not be considered detrimental only based on a test of chi-square when determining the overall validity of the model, and other indicators should be taken in consideration [18].

Overall, the results of the proposed APUTCM scale and CCTCM scale research models show acceptable fits. The scales that were used in this study were the best fit to the data, within a desirable range. Both scales also included their separate latent factors under the higher order latent factors.

### Convergent & divergent validity

The convergent and divergent validity for establishing construct validity were determined by the associations between scales and other variables, such as sex, age, education level, years of experiences, job position, frequency of journal readings, self-use TCM, TCM education status, self-perceived knowledge of TCM, self-perceived communication of TCM, TCM practice, and frequency of TCM practice. Table 3 shows the means and standard deviations for each of the two groups for each scale. There were no significant differences between males and females, which indicates no sex-biased attitudes in this scale. A strong relationship was found between scale and participant characteristics, such as job position, self-use TCM, and TCM practice (Table 3). A strong relationship between the APUTCM and CCTCM scales was observed (γ^2^ = .777). Both scales were significantly associated with age, education level, years of experience, frequency of journal reading, self-perceived knowledge of TCM, self-perceived communication of TCM, and frequency of TCM practice (Table 4). The discrimination validity for both scales was tested with respect to the differences that were observed between nurses with and without TCM education. There were significant differences between the two groups in the scores of the APUTCM scale (*t* = − 7.92, *p* < .001) and the CCTCM scale (*t* = − 4,87, p < .001).

**Table 3:** Relationships between variables and scales

Scales/Variables	Groups	APUTCM				CCTCM			
		mean	SD	t	*p*	mean	SD	t	*p*
Gender	Male	59.75	8.00	−1.33	.185	22.33	3.06	−1.15	.250
	Female	64.23	11.65			24.15	5.45		
Position	RN	63.84	11.76	−2.27	.023	23.91	5.47	−3.17	002
	NP	67.03	9.89			25.98	4.681		
Self-use of TCM	No	61.40	11.32	−9.52	.000	23.14	5.32	−7.04	000
	Yes	69.43	10.30			25.99	5.14		
Received TCM education	No	61.78	11.59	−6.98	.000	23.33	5.43	−4.87	.000
	Yes	67.67	10.83			25.27	5.24		
Practice of TCM	No	62.44	11.43	−9.60	.000	23.51	5.41	−6.24	.000
	Yes	71.24	9.32			26.61	4.68

**Table 4:** Correlations between variables and scale

Scales	APUTCM	Age	Education level	Years of experiences	Frequency of journal readings	Perceived knowledge of TCM	Frequency of TCM communication
APUTCM							
γ^2^	1	.169	.141	.153	.109	.317	.297
*p*		.000	.000	.000	.003	.000	.000
CCTCM							
γ^2^	.817	.172	.143	.128	117	.273	.266
*p*	.000	.000	.000	.000	.001	.000	.000

## Discussion

This scale development study provides preliminary evidence supporting the reliability and validity of the 13-item APUTCM scale and the five-item CCTCM scale assessing the attitudes of nurses’ towards patient’s TCM use and communicative competence in TCM, as the findings show good internal consistency and strong construct validity. After removing the non-consensus items from both scales, the findings showed good content validity determining through reviewer feedbacks and professional judgements. For reliability, the scales showed evidence of acceptable internal consistency, which indicates that the corresponding items measured the same concept, with no indications of item redundancy (correlations ranged from 0.67 to 0.84). The SEM of data from both scales demonstrated substantial support for construct validity. The convergent and divergent validities of the scores from both scales provide evidence in the expected direction.

Three factors were derived from the APUTCM scale as a result of the exploratory factor analysis, which was consistent with the tri-component attitude model [14]. The scale combines cognitive, affective, and behavioural dimensions that measure nurses’ attitudes towards patient’s TCM use. Most measurement scales for “attitudes” in literature have been developed to primarily emphasize attitudes towards TCM or each TCM therapy [6, 19], and such scales seldom measure attitudes towards patient’s TCM use. The cognitive component of the APUTCM scale refers to the beliefs and opinions of nurses toward patient’s TCM use. The affective attitude component captures nurses’ direct or inclusive assessment of their affective response to patient’s TCM use. The behavioural component refers to the attitude of nurses with regards to patient’s TCM use. The reliability and validity findings of the APUTCM scale demonstrate efficient and robust model fitting with the essential model parameters. The scale items describe specific beliefs, opinions, and actions that provide guidance for promoting the communication of TCM with patients.

The CCTCM scale reflects nursing behaviour and directly assess how confident nurses regard TCM communication with patients. Two factors emerged from the scale as a result of the exploratory factor analysis, sustainability and performance. The sustainability of the CCTCM scale refers to the ability of maintaining a certain level of TCM communication skills. The performance of the CCTCM scale indicates the capacity of nurses to communicate TCM issues with patients. Only, the performance subscale of CCTCM showed slightly lower internal consistency, which may have been due to the small number of items and the nature of the items that composed the scale [20]. To our knowledge, there is no such scale to assess the competence of TCM communication.

For convergent validity, strong correlations were found between the APUTCM and CCTCM scales and both scales showed significantly positive relationships with the following indicators: age, education level, years of experiences, frequency of journal reading, self-perceived knowledge of TCM, self-perceived communication of TCM, and frequency of TCM practice. The discrimination validity and participant characteristics, such as nurse practitioners having better APUTCM and CCTCM scores than nurses, participant self-use of TCM, prior TCM education, or TCM practice in clinical care were associated with higher APUTCM and CCTCM scores. The findings of this study were similar to those of a previous study, in which participants with knowledge about TCM had a significantly (*p* < 0.05) higher odds of responding to patient questions about TCM use (OR 3.3; CI 1.4–7.6) [5].

The reliability and validity findings of the APUCAM and CCTCM scales demonstrate efficient and robust model fitting with the essential model parameters. The items of APUCAM scale describe one’s beliefs, opinions, and actions on patient’s TCM use with higher scores indicating more positive attitudes which provide guidance for promoting the communication of CAM with patients. The items of CCTCM scale describe one’s ability, capacity, skill in TCM communication with higher score indicating a better performance. Based on the WHO Traditional Medicine Strategy: 2014–2023, a culture of communication is encouraged not only amongst healthcare professions but also between patients and healthcare professionals [1]. Competence requires knowledge and the right attitude that eventually transforms to behaviour or skills [21]. Therefore, building an attitude is the first step of being competence. The more positive attitudes nurses have, the greater performance of TCM communication nurse can be. Given the fact, nurses are often the first point of contact and are involved with the longest period of care for patients within the healthcare system. In any hospital visit, history-taking and health assessment are crucial components for further diagnosis and treatment management. Nurses should perceive their role as gatekeepers by undertaking the first assessment of patients related to TCM use within the healthcare practices as well as accompanying patients through the integration of conventional medicine and TCM.

This study has several limitations. First, Chi-square goodness of fit test for the models were significant, which indicates an imperfect fit. However, the chi-square statistic is very sensitive to sample size and is not an appropriate indicator of fit in large data sets alone. To evaluate the fit of structural equation models, the ratio of Chi -Square/df (< 5) is recommended as a basis for acceptance or rejection [17]. Therefore, the two models of chi-square/df results in this study are 3.891 and 4.04 which is acceptable. Second, the generalizability of this study may be limited to other countries, as the sample largely comprised Taiwanese individuals. In addition, the percentages of male nurses in Taiwan vs. male participants in this study are 0.028 vs. 0.015 respectively [22]. Using Chi-square with Yates correction resulted in the two-tailed *p* value >.05. It is considered to be not statistically significant differences between two groups. However, further research should explore this measure in other countries with the consideration of sex-ratio, and additional validations should be performed, including additional convergent and divergent validity tests.

## Conclusions

This study was to develop and validate attitude scales towards patients’ use of TCM (APUTCM) and communicative competence scale in TCM (CCTCM) among nurses. The both scales showed appropriate reliability and validity, including internal consistency, content validity, construct validity, convergent validity and discrimination validity. The APUTCM scale can be used to assess the cognitive, affective, and behavioural components of attitudes towards patients’ TCM use. The CCTCM scale determines accountability and performance competence when communicating with patients about TCM. Given current prevalence of CAM use and concerns about the safety of TCM use, it is no longer acceptable to disregard TCM in patient care. Communication about TCM use between patients and nurses is the key to ensuring the safe implementation of integrated TCM and conventional medicine. Understanding nurses’ attitudes towards patients’ TCM use and nurses’ competence in TCM communication may gain more insight into their attitudes and abilities to do the task successfully or efficiencily. It could help to build the professional continuing education on how to create a safe and care environment and let an open dialogue clarify expectations and share the decision-making of TCM use between patients and nurses to ensure that patients use TCM safely.

## Data Availability

All relevant data are included in this manuscript.

## Abbreviations

APUTCM: Attitudes towards patients’ use of TCM
CCTCM: Communicative competence in TCM
TCM: Traditional and Complementary medicine
